# Land system change and food security: towards multi-scale land system solutions^☆^

**DOI:** 10.1016/j.cosust.2013.07.003

**Published:** 2013-10

**Authors:** Peter H Verburg, Ole Mertz, Karl-Heinz Erb, Helmut Haberl, Wenbin Wu

**Affiliations:** 1 Institute for Environmental Studies (IVM), VU University Amsterdam, de Boelelaan 1087, 1081 HV Amsterdam, The Netherlands; 2 Department of Geosciences and Natural Resource Management, University of Copenhagen, Øster Voldgade 10, 1350 Copenhagen K, Denmark; 3 Institute of Social Ecology Vienna (SEC), Alpen-Adria Universitaet (AAU), Schottenfeldgasse 29, 1070 Vienna, Austria; 4 Institute of Agricultural Resources and Regional Planning, Chinese Academy of Agricultural Sciences, Beijing, China

## Abstract

Land system changes are central to the food security challenge. Land system science can contribute to sustainable solutions by an integrated analysis of land availability and the assessment of the tradeoffs associated with agricultural expansion and land use intensification. A land system perspective requires local studies of production systems to be contextualised in a regional and global context, while global assessments should be confronted with local realities. Understanding of land governance structures will help to support the development of land use policies and tenure systems that assist in designing more sustainable ways of intensification. Novel land systems should be designed that are adapted to the local context and framed within the global socio-ecological system. Such land systems should explicitly account for the role of land governance as a primary driver of land system change and food production.

## Highlights

• Land system science plays a key role in the food security challenge.
• Land availability and intensification potential require a nuanced assessment.
• The role of land tenure and governance systems in food supply requires more attention.
• Increased food production requires both agronomic innovations and the design of novel land systems.

**Current Opinion in Environmental Sustainability** 2013, **5**:494–502

This review comes from a themed issue on **Human settlements and industrial systems**

Edited by Peter **H Verburg, Ole Mertz, Karl-Heinz Erb** and **Giovana Espindola**

For a complete overview see the Issue and the Editorial

Available online 1st August 2013

1877-3435/$ – see front matter, © 2013 The Authors. Published by Elsevier B.V. All rights reserved.

**http://dx.doi.org/10.1016/j.cosust.2013.07.003**

## Introduction

The challenge to produce about 70% more food by 2050 for a growing and increasingly affluent world population has motivated several review papers addressing the different dimensions of the food system and food security [1–7]. Food security is determined by food availability (the overall production of food by the agricultural system), food access (distributional and entitlement issues), food stability (the risk of losing temporarily or permanently access to food), and food utilization (all food safety and quality aspects) [8,9]. Land-based production provides the major biophysical basis for food security. Land system change, that is, the spatial and temporal changes in the interplay of social and ecological systems in shaping land use and land cover, is central to food security assessments. Land system change may originate from the increasing demands for land-based products, or from competition for land resources, not only to produce food but also to provide materials and feedstock for the bio-based economy, for nature conservation, urban development and recreational facilities [10^•^,11,12,13,14]. While different authors emphasize different dimensions of the food system, there are many scholars who argue that increases in food supply will have to rely on sustainable intensification, that is, agricultural practices that allow for yield increases without negative consequences for the social and ecological conditions [7]. The large increases in agricultural production over the recent decades are mainly due to intensification, while area expansion was limited [15,16]. There is still only a limited potential for cropland expansion and such expansion would cause losses to (semi-)natural ecosystems with manifold detrimental socio-ecological consequences. Conservationists have praised ‘sustainable intensification’ for its potential land sparing effects [17]; others have nuanced the notion of ‘sustainable intensification’ by de-coupling the terminology from large-scale farming and pointing at the location specific, negative externalities of such intensification [18^•^,19].

Notwithstanding the enormous success of the green revolution in increasing agricultural production through intensification, there is a widespread acknowledgement that meeting the food security challenge requires more than agronomic research and technological innovation to increase yields. Several authors have argued in favour of a food systems approach [4,5,20]. Access to food is not the only relevant socio-economic dimension of food security. Food security is also intricately linked to land governance [21,22], dietary choices [6,23,24], agricultural policies [25] and environmental perceptions and attitudes [26^•^], among others.

Rather than repeating the arguments made in the large series of review papers on food security issues that have been published recently, we will discuss five areas in which land system science can make a contribution to scientific analyses of food production, as an important component of food security. These include: (a) land available for expansion of food production areas, (b) potentials to increase agricultural yields, (c) interactions between local solutions and the global context, (d) the role of land governance, and (e) the potential of land system architecture. Based on the reviewed literature and discussions within the research community, these are considered highly important areas where land system science can help to substantiate the food security debate and contribute to the design of pathways to meet the food security challenge. Each topic is addressed by reviewing the recent literature and discussing the challenges for land system science. The concluding section provides a framework connecting the different approaches in land system science to address the challenges identified.

## Land as a scarce resource: the myth of ‘available’ land

An obvious starting point for land system science is the assessment of land available for expansion of agricultural production areas [12]. Available land has been addressed in particular in the context of the land demand for bioenergy production [10^•^,27–32]. Many assessments of land availability exclude forest lands and focus instead on so-called ‘unused’ and ‘degraded’ lands, sometimes addressed as wastelands. The focus on ‘unused’ and ‘degraded’ lands assumes that strong governance of the remaining forest resources is feasible. In practice, forest lands may be more attractive for agricultural development and other available land resources may be overlooked [33]. Excluding forest land but including grasslands, secondary vegetation and other (semi)natural land cover types in assessments of farmland availability is based on a tacit assumption that forests are more valuable than other ecosystems which may not necessarily be the case.

The large variation in estimates is illustrated by the range between low estimates of 52 Mha for both agricultural production and biofuels [34] to high estimates of 1107 Mha, that include marginal land that can be cultivated for biofuel production [35]. The large variation in estimates of available land are not only a result of different assumptions of what land can be considered as available and for what purpose [29], also differences and errors in global datasets of land use and relevant attributes, for example, the degradation status of lands, are a large source of uncertainty [36,37]. Areas deemed available for agriculture are often areas with a land cover (and use!) that cannot be easily classified into one of the commonly used land classification systems to make land cover inventories from remote sensing, and this can easily lead to politically motivated classifications [38,39]. In many cases, land classified as ‘unused’ or ‘degraded’ is in fact used for extensive livestock grazing or shifting cultivation, often by subsistence farmers or herdsmen [39,40], and in many developing countries secondary vegetation is an important source of forest products and biodiversity [41]. Conversion of land for food production always has trade-offs in terms of the ecosystem services provided to society. In addition, ownership can inhibit the conversion of these lands to agricultural use and thereby limit the effective land availability.

Another reason for overestimating land availability is related to the approaches underlying the estimates. In such analyses, the full area of a polygon or pixel is often counted as being available for agricultural use. Due to the space required for infrastructure (e.g. for drainage, irrigation and housing) and the presence of natural and cultural landscape elements (e.g. rock outcrops, wetlands, hedgerows) less than the full surface area can actually be cultivated [29,42]. Over time, available land is expected to further decrease due to losses of productive agricultural lands to urban development and associated land uses such as infrastructure and recreation (e.g. parks, golf courses). Due to the historic location of cities in the middle of fertile agricultural lands or at strategic locations along rivers and the coast, urban development often coincided with the location of the best agricultural lands [43] and despite long term settlement, these areas can even be hotspots of biodiversity and conservation efforts may further reduce the availability of land [44]. Climate change poses another threat to agricultural areas by increased flooding of agricultural lands near rivers as well as increasing occurrences of droughts [45^•^,46]. At the same time, more lands previously unavailable to agricultural use can be taken into production due to more favourable climatic conditions [47].

Taken together, there is probably less land available for additional farmland than top-down assessments indicate. Furthermore, in many cases, converting additional land is associated with trade-offs. At specific locations, the tradeoffs of converting some of the ‘unused’ or ‘degraded’ lands to agricultural production may be acceptable and restoration of ‘degraded’ land by a combination of afforestation and agricultural production may have significant potential [48]. If properly designed and managed, well-adapted farming systems may reduce further degradation and eventually increase the provision of selected ecosystem services [49].

By simply classifying lands as available we may overestimate the production potential and underestimate the trade-offs of such a strategy. Assessments of land availability should account for these trade-offs and limitations. As land availability estimates also play an important role in dynamic models of food supply [50^•^,51,52], a more nuanced assessment of land availability is an important research priority.

## Closing the yield gap requires an in-depth understanding of the driving factors of intensification

The large potential for improving production through intensification has been frequently mentioned in the literature [2,6,53^•^,54,55]. The yield gap indicates the difference between yield potential and the average farmers’ yields over some specified spatial and temporal scale of interest [56]. Yield potential is defined and measured in a variety of ways, often referring to the attainable yield (the yield that can be achieved at a location under optimal management). At the global scale, the analysis of the yield gap is either based on comparing crop growth models with actual yields as reported in statistics [57] or based on empirical techniques that compare highest achieved yields with actual yields reported in the same database to avoid bias due to the use of multiple, inconsistent data sources [53^•^,54]. There are, however, many reasons for deviations between actual and attainable yields as well as between actual and potential cropping cycle intensity (i.e. the length of fallow periods and number of crops cultivated per year) [53^•^,58]. Diminishing returns on inputs may be one reason, as it reduces the economic profitability of increasing inputs [59,60], although rising prices of agricultural commodities will make increased use of inputs more profitable. There are many other reasons for non-optimal management of croplands. These include limited access of land managers to credits, limited access to agricultural inputs and knowledge, and high risk of losses on crop failure due to climate variability or change. In some cases, sub-optimal inputs are related to land governance and tenure, for example, as a result of insecure property rights. Conversely, tenure does not always guarantee higher investments [61,62]. In many cases the yield gap strongly differs for rainfed and irrigated agriculture [56]. Irrigation is, therefore, a requirement for intensification in many areas, in particular, but not constrained to drylands. However, irrigation often requires central investments in infrastructure [63] and often water availability for irrigation is limited [64]. Closing the yield gap often comes at the cost of environmental and social externalities, for example, pollution of surface and groundwater by agro-chemicals and loss of employment due to mechanization [65]. The ecological costs of closing the yield gap can be minimized by focusing efforts to increase yields in regions with the lowest yields, which requires global efforts for technology transfer from rich to poor countries. Such a strategy would enhance the soil fertility in these regions while using fertilizer inputs efficiently [7].

The underlying reasons for the yield gap and the implications of intensification have mostly been analysed in case studies [66]. Further research is needed to match the understanding of the constraints to closing the yield gap at the local level to the level of global assessments. This will help to identify regions where different solutions (technical, infrastructural or socio-economic) may alleviate the constraints for intensifying production with relatively low environmental and social externalities. Pretty *et al*. [67] report a meta-analysis of case studies of sustainable intensification options as applied in 286 projects worldwide. The results show that, in spite of high variation in success, there is a prospect to increase agricultural production without increasing environmental externalities. The meta-analysis particularly mentioned measures for more efficient water use in both dryland and irrigated farming, improvements in organic matter accumulation in soils and pest, weed, and disease control emphasizing in-field biodiversity as measures that yielded both positive impacts on yield and an improved supply of ecosystem services. Thus, sustainable intensification is indeed an option, but requires full consideration of the socio-economic and environmental drivers underlying current non-optimal yields.

## Interactions between local solutions and the global context

Local visions of agricultural development can lead to important deviations from the overall regional or global trend towards intensification and scale enlargement. As a response to large-scale intensification based on high inputs of chemicals and globalization of food markets, organic agriculture, local products and urban agriculture have emerged as more localized alternatives. The food security debate has been influenced by strong opinions, even dogmas, in favour of or against such developments [68–72]. In these discussions, organic agriculture has often been characterized by yield levels that are far below those of conventional agriculture and consequently requiring a much larger production area than conventional agriculture, and thus, not being capable to fulfil the global food demand [73]. Two recent meta-analyses of case studies comparing organic and conventional agriculture [74,75] suggest that organic yields are, on average, indeed lower than in conventional systems. However, the same meta-analyses indicate that under specific conditions, organic systems can nearly match conventional yields. At the same time, intensive agriculture has been characterized by negative environmental and social externalities. Pretty *et al*. [67] have, however, shown that sustainable intensification may be possible and effective, depending on the local context.

The above indicates the importance of local context in discussions of ways to increase food production in a sustainable manner. In some places, organic agriculture or other extensive, multifunctional, forms of agriculture can be a suitable option, for example, in sensitive environments. At the same time, intensive agriculture can, with the right technology and management, produce large quantities of commodities fulfilling the food demand of many people on a relatively small area. While urban agriculture and local production networks are unlikely to make large contributions to matching demand for food production on a global scale, they may provide increased local access to food, fulfil niche markets and provide important other functions, such as social cohesion and education [69]. Matching land use systems with the abilities and willingness of the land managers, the local environmental conditions and the demand for ecosystem services is important to achieve sustainable land management.

However, in a globalized world the wrong conclusion may be drawn if only the local context is accounted for [76]. Choices for a specific production system always have global impacts: the choice for relatively unproductive systems has trade-offs due to displacement of production to other places [10^•^]. At the same time, it would be incorrect to assume that intensification will always spare land that can be used for conservation purposes. Increased production can trigger increased consumption as result of lower prices and improved agricultural opportunities may attract new activities on ‘spared’ land [10^•^,19,77^•^,78].

Highly productive systems situated far from consumers are vulnerable to wasted food during transportation. Optimization of food production in those areas that are best suited for intensive production with low environmental externalities can be favourable unless the increased environmental pressures resulting from transportation and trade off-set the benefits [79]. However, in many cases greenhouse gas emissions related to production are much larger than those resulting from transport, distribution and storage [80]. Shifts towards locally produced food only helps in reducing emissions as long as savings of transport emissions are not compensated by increased production emissions [81,82]. On the other hand, for some grains (esp. rice) global markets are small compared to domestic markets [83], many have poor access to markets and many countries do not want to be strongly dependent on other countries for their food security. The contributions of local solutions to meet regional and global food demand, therefore, need to be better understood and analysed in relation to the need for closing yield gaps and promoting sustainable intensification as discussed above.

## Understand the role of land governance as a key determinant of land systems and food production

The limits of analyzing and modelling the land system based on assumed economically rational behaviour and perfect land markets are well known [26^•^,84]. One of the main aspects insufficiently addressed relate to land governance. Conceptual models of property rights and institutions often ignore that, in many instances, the distribution of land ownership is not the result of the operation of “perfect” markets but reflects power structures. Land registration can stimulate farmer's control on the lands they manage. However, it may also be viewed as an attempt to cement existing unequal arrangements rather than to overcome them [62]. Land rights or tenure provides the owner with a secured capital for production and the distribution of land rights has, for example, in the case of China, led to increased investments by farmers. Here, the permission of transferring the land rights to others for limited periods of time has given rise to scale enlargement and intensification [85,86]. In Eastern Europe the distribution of land rights has had mixed impacts on production, especially leading to increased fallow and abandonment of previously productive agricultural areas [87]. Moreover, the role of land rights and governance of land resources has, in the past few years, received increasing interests due to the large areas that are subject to land acquisition by large companies from inside or outside the country [22,88]. While such developments may have positive impacts on food production due to capital investments, much of the production is shipped abroad and sometimes land is just left fallow for speculative purposes [89,90].

Overall, there is little general insight into the role of institutions, land tenure and land governance on land use intensity as most research is based on case studies that are strongly context dependent [62]. The fact that a very large part of the land used for food production is privately owned or has been allocated long term land rights to private persons or companies makes the top-down implementation of improved land system management that better match food production demand and ecosystem protection impossible. On the other hand, centralized governance of land management and intensification has had disastrous consequences in a number of well documented cases [91,92]. In the end, most land use decisions are the result of the behaviour of land owners and land managers that responds to market prices and policy incentives in varying ways. There is, thus, an urgent need to better understand how land rights and land governance influence land management decisions and the implementation of new land use systems. Alternative modes of land governance need to be investigated that allow more responsiveness to the tradeoffs involved in land use decisions. One of those directions is research on the role of certification of production systems. Balmford *et al*. [93] suggest that such schemes might be realigned away from rewarding low-yield farming towards incentivizing producers who instead set aside areas of land for conservation. However, there are also concerns that incentives for conservation or ecosystem service provision, including REDD+, may not have the theorized positive effect on food security as they imply an intensification of agriculture that is not at the core of the payment scheme [94]. Understanding the ways in which novel incentives to local farmers can be used to achieve sustainable production systems requires more attention to collectively steer land management towards more sustained practices than can be ensured by market drivers alone [95,96].

## Land systems architecture and prototyping

One of the ‘grand challenges’ for sustainability science identified by Reid *et al*. [97] is the need to co-design research that addresses societal problems and produces implementable knowledge in collaboration with stakeholders. This notion is also core to the recently launched new international coordination of global change research: Future Earth (http://www.icsu.org/future-earth). For land system science this requires a stronger focus on the participatory design and implementation of novel land use management strategies.

Solution oriented research in the field of food production is now mostly focused on crop and animal breeding and agronomic technology [98]. The high returns on investments in such research projects in both developed and developing countries are an indicator of their success [99]. However, a perspective beyond crop breeding and farm technology is needed, extending to the whole land system. Sustainable intensification requires the design of new production systems that are able to produce more food and ecosystem services demanded by society, while being resilient and adaptive to societal and environmental changes. This requires the development of new farming systems, especially novel ways of integrating livestock and arable systems and possibly the integrated production of food and energy, taking into account the local needs and expectations in a participatory approach. In particular, it requires the design of land use systems beyond the farm level, integrating the farming activities within the structure and composition of the landscape as a whole. Such design, called land systems architecture [100^•^], aims at optimally using the spatial and temporal structure of the land system to produce ecosystem services, provide resilience against disturbances and reduce risks. The heterogeneity of landscapes ensures that different land uses are not necessarily competing for the same land areas within the landscape as each function has specific requirements in terms of climate, soil, moisture, and terrain characteristics. This provides the possibility to design land systems that combine different functions in which the multiple demands of society can be fulfilled by the landscape system as a whole [101]. Such land systems architecture should not aim at the design of one optimal system that can be implemented worldwide, but rather aim at systems that fit the local context, including aspects of land governance and jurisdiction. This requires a stronger integration of socio-ecological systems analysis, land use planning and agronomic research into an integrated approach that co-designs novel land systems in close collaboration with the local stakeholders while accounting for the regional and global context.

## Conclusions: the role of land system science in food security research

The discussion has shown that land system science covers central aspects of the food security debate. However, the food security challenge is much broader than food production systems alone. All too often the need to supply 70% more food is quoted; some authors even foresee a doubling of demand in the next decades [7], based on studies by the FAO and World bank using strong, debated, assumptions [102^•^,103]. The enormous challenge of reaching such increases and the unavoidable tradeoffs with other services and biodiversity, also requires efforts on reducing the need to increase production by limiting food waste [104,105] and lowering demands by dietary change [6,23]. It is widely acknowledged that de-coupling increasing economic prosperity and urbanization from increased meat consumption is difficult, as the inelastic demand makes taxing of these products inefficient. In spite of these difficulties, such options should not be ignored as changes in behaviour and lifestyles provide an enormous potential to reduce the food supply challenge. Furthermore, not all additional demand for food production has to be supplied by the land. Although the scope for increased harvesting of fish is limited [106] the capacity of the oceans to produce algal biomass should not be underestimated [107]. Too easily, the food challenge is translated into an agronomic challenge out-ruling other important options. To assist in evaluating alternative options, Computable General Equilibrium models are very useful tools to help explore feedbacks between demand and supply and evaluate the net effect of different strategies and policy instruments [50^•^,108,109].

**Figure 1:**
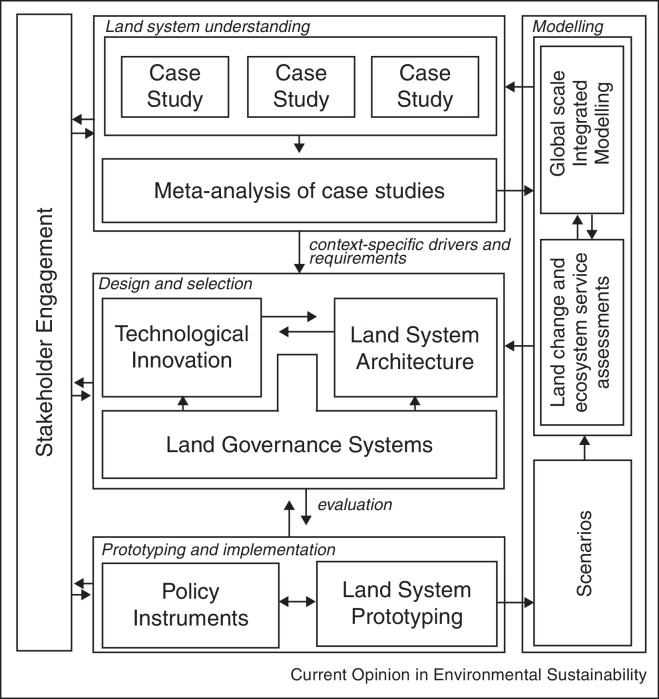
Research framework for land science contribution to food security research.

Meeting the challenges outlined in this paper requires integrative science across scales, disciplines and new partnerships beyond the scientific community. An overview of the required land system science components is shown in Figure 1. We not only need to contextualise local research and local solutions in regional or global frameworks, but also improve our global assessment tools with information from local case studies. Meta-analytic approaches to distinguish those factors that are generic and those that are case-specific are useful tools to synthesize case study findings into inputs to model design and parameterization. Too often the lack of social science data and the barriers of integration of social sciences with natural sciences cause important social drivers and land governance processes to be ignored in global assessment frameworks. In selecting case study locations and topics, we need to reduce bias in selection procedures. Balmford [93] indicates that in farming system studies some of the major crops and livestock systems are largely underrepresented. In land science, meta-analytic studies often focus on drivers of land cover change [110,111], whereas far fewer studies document the drivers of agricultural intensification [66]. Such bias in research is common across many disciplines [112]. However, it is a luxury of science that cannot persist under the pressing need to better understand food production and agricultural intensification processes. Targeted case studies and their synthesis that address the interaction between socioeconomic and natural factors in intensification are needed across all major land system types and processes of change. Such knowledge will help to improve the representation of land change processes, feedbacks and decision making in large scale land change models that currently often rely on oversimplistic representations of the diversity in decision making upon changes in drivers. At the same time, novel ways of linking local case studies with global drivers and feedbacks as determinants of land use decisions and impacts are required.

Land system architecture requires a stronger link between explorative research and design-based approaches [100^•^]. Model based assessment of alternative land systems and prototyping such systems will help test the function of novel land systems and provide exemplars of practice and innovation [113,114].

The sum of these scientific challenges places land system science — as interdisciplinary, problem-oriented and cross-scalar endeavour — in an excellent position to integrate various research activities and thereby contribute to the establishment of robust strategies towards tackling the food security challenge.

## References and recommended reading

Papers of particular interest, published within the period of review, have been highlighted as:

• of special interest
•• of outstanding interest
